# Production of interleukin-17 in Behcet’s disease is inhibited by cyclosporin A

**Published:** 2010-05-19

**Authors:** Wei Chi, Peizeng Yang, Xuefei Zhu, Yuqin Wang, Lina Chen, Xiangkun Huang, Xiaoli Liu

**Affiliations:** 1 Zhongshan Ophthalmic Centric, Sun Yat-sen University, Guangzhou, P.R. China; 2 State Key Laboratory of Ophthalmology, Sun Yat-sen University, Guangzhou, P.R. China; 3 The First Affiliated Hospital of Chongqing Medical University, Chongqing Key Laboratory of ophthamology, Chongqing Eye Institute, Chongqing, P.R. China; 4 Department of Ophthalmology, The First Affiliated Hospital of Suzhou University, Suzhou, P.R. China; 5 Eye Hospital of Wenzhou Medical College, Wenzhou, P.R. China

## Abstract

**Purpose:**

Behcet’s disease (BD) is a systemic inflammatory disease presumably caused by an autoimmune response. Interleukin (IL)-17 has been demonstrated to be involved in the development and maintenance of certain inflammatory diseases, including BD. This study was designed to investigate the influence of cyclosporine A (CsA) on IL-17 production by peripheral blood mononuclear cells (PBMCs) from BD patients in vitro and in vivo.

**Methods:**

Fifteen BD patients with active uveitis were involved in this study. Blood samples were taken from these patients for analysis of IL-17 and interferon (IFN)-γ. Six patients were re-evaluated at 1 and 3 months after treatment with CsA. The levels of IL-17 and IFN-γ in the supernatants of PBMCs from patients before treatment cultured without or with CsA at different concentrations were detected by enzyme-linked immunosorbent assay (ELISA). Flow cytometry was used to evaluate the frequencies of IL-17-producing and IFN-γ-producing T cells and the expression of CD69 on CD4^+^ or CD8^+^ T cells before, 1, and 3 months after CsA treatment.

**Results:**

The results showed that significantly higher levels of IL-17 and IFN-γ were observed in active BD patients as compared with controls. Treatment with CsA could inhibit the production of both cytokines in association with an amelioration of intraocular inflammation. In vitro, CsA significantly inhibited the production of IL-17 and IFN-γ by PBMCs activated with anti-CD3 and anti-CD28 antibodies or phorbol 12-myristate,13-acetate and ionomycin in BD patients with active uveitis. However, CSA did not influence the CD69 expression in CD4^+^ and CD8^+^ T cells induced by phorbol 12-myristate,13-acetate (PMA) ionomycin.

**Conclusions:**

Our findings showed that CsA can significantly inhibit the intraocular inflammation of BD patients and the expression of IL-17 and IFN-γ in vivo and in vitro. The results suggested that the inhibitory effect of CsA on uveitis in BD patients may be partially mediated through inhibiting the production of IL-17 and IFN-γ.

## Introduction

Recent studies have found a new subset of CD4^+^ T helper (Th) cells that selectively produce interleukin (IL)-17 and play a critical role in the pathogenesis of autoimmune and chronic inflammatory disorders [1]. IL-17 is a 17-kDa protein, secreted as a disulfide-linked homodimeric glycoprotein, and is a member of the IL-17 family [2]. Several reports have shown that IL-17 stimulates the induction of various pro-inflammatory cytokines and chemokines [3,4]. Accumulating evidence suggests that several inflammatory and autoimmune diseases in human and mouse, such as rheumatoid arthritis, multiple sclerosis, Crohn’s disease, psoriasis, and uveitis, are associated with IL-17 overexpression and production [5-10].

Behcet’s disease (BD) is a chronic, systemic, relapsing inflammatory disease mainly showing as four major manifestations: recurrent uveitis, oral aphthae, genital ulcers, or skin lesions [11]. Although various etiologies have been presumed, BD is believed to be an autoimmune disease in origin [12-14]. Our recent study showed that IL-17 was upregulated in BD patients with active uveitis as compared with BD patients with inactive uveitis and healthy individuals [10].

Cyclosporine A (CsA) has been shown to be effective in reducing the frequency and severity of BD, especially intraocular inflammation [15]. It has been demonstrated that CsA could inhibit the production of several inflammatory cytokines, such as IL-12, IL-18, and tumor necrosis factor-α [16,17]. Several reports have shown that CsA could inhibit IL-17 production in certain autoimmune diseases, such as Vogt-Koyanagi-Harada (VKH) syndrome [18-20]. It remains unclear whether CsA can also exert its function via inhibiting IL-17 production in BD. The purpose of this study was to investigate the effect of CsA on the expression of IL-17 in BD, in vivo and in vitro. The results showed an increased production of IL-17 and interferon-γ (IFN-γ) by peripheral blood mononuclear cells (PBMCs) in BD patients with active uveitis. In vitro and in vivo experiments revealed that CsA significantly downregulated both IL-17 and IFN-γ expression in active BD patients. These results suggest that CsA may inhibit the intraocular inflammation of BD, presumably by suppressing both IL-17 and IFN-γ production.

## Methods

### Patients

Fifteen BD patients with active uveitis (nine men and six women), with an average age of 36 years, and 14 healthy individuals (nine men and five women), with an average age of 35 years, were included in this study. All study subjects were recruited from Zhongshan Ophthalmic Center, Sun Yatsen University (Guangzhou, P.R. China) from April 2007 to January 2009. The diagnosis of BD disease was based on the diagnostic criteria designed by the International Study Group for BD disease. In brief, the diagnostic criteria include the presence of recurrent oral ulceration plus two of the following: recurrent genital ulceration, eye lesion (anterior or posterior uveitis), or skin lesions (erythema nodosum, pseudofolliculitis or papulopustular lesions) [21]. All of these BD patients showed active recurrent intraocular inflammation, evidenced by keratic precipitates (100%), flare and cells in the anterior chamber (100%), vitreous cells (46.7%), and retinal vasculitis, observed clinically or disclosed by fluorescein angiography (100%). The extraocular manifestations were recurrent oral aphthous lesions (100%), multiform skin lesions (66.7%), recurrent genital ulcers (44.4%), and arthritis (33.3%). Six out of these 15 patients had been intermittently treated with corticosteroids for at least 1 year before coming to the Zhongshan Ophthalmic Center, Guangzhou, P.R. China. However, these patients responded poorly to steroid therapy. All of the 16 patients did not use immunosuppressive agents for at least 1 week before visiting us. Blood samples were collected by veinpuncture from all of the 15 patients before in vitro and in vivo treatment and normal controls. The heparinized tubes, containing blood samples, were immediately transferred to the laboratory, and then PBMCs were isolated from blood samples by Ficoll–Hypaque density-gradient centrifugation to be subjected to the following experiments. After blood sampling, CsA (3.0–5.0 mg/kg per day) in combination with oral prednisone (15–20 mg/day) was used for the aforementioned six patients. These six patients were re-evaluated at 1 and 3 months after CsA in vivo treatment. The experimental design is described below in detail. The ocular manifestations of the six patients before and after treatment with CsA are summarized in Table 1. Fourteen age- and sex-matched, untreated, healthy individuals, without ophthalmic diseases, were included as control group. All patients and healthy controls showed no associated systemic abnormalities, and had normal psychomotor development at the time of collecting blood samples. All procedures followed the tenets of the Declaration of Helsinki, and informed consent was obtained from all patients and normal controls.

**Table 1:** Ocular manifestations of BD patients before and after CsA treatment.

** **	**KPs**			**Aqueous cells**			**Aqueous flare**			**Vitreous cells**		
**Patient number**	**0**	**1**	**3**	**0**	**1**	**3**	**0**	**1**	**3**	**0**	**1**	**3**
1	+++	++	±	+++	++	+	+++	++	+	++	+	±
2	+++	+	−	++	+	−	+++	+	+	−	−	−
3	+++	++	−	++	+	−	++	+	−	−	−	−
4	++	+	−	++	+	−	++	+	−	+	±	−
5	+++	++	±	+++	+	±	+++	++	±	++	+	+
6	++	+	+	++	+	−	++	+	−	−	−	−

In the second row, the “0” indicates before CsA treatment, the “1” indicates after 1-month CsA treatment, and the “3” indicates after 3-month CsA treatment.

### Measurement of cytokines by enzyme-linked immunosorbent assay

PBMCs were stimulated with or without anti-CD3 (5 µg/ml; eBioscience, San Diego, CA) and anti-CD28 antibodies (1 µg/ml; eBioscience) for 72 h for determination of IL-17 and IFN-γ production in BD patients before and after CsA treatment. The supernatants were then collected for analysis. IL-17 and IFN-γ levels were detected using the Duoset enzyme-linked immunosorbent assay (ELISA) Development kit (Research and Diagnostics System, Minneapolis, MN), with a detection limit of 15 pg/ml. To determine the effect of CsA on the production of IL-17 and IFN-γ production in vitro, different concentrations of CsA (0.4, 2, 10, 50, and 100 ng/ml; Novartis, Switzerland) were added to cultured PBMCs stimulated with anti-CD3 and anti-CD28 antibodies for 72 h.

### Intracellular cytokine staining

PBMCs were stimulated with 20 ng/ml PMA and 1 μg/ml ionomycin (Sigma-Aldrich, St. Louis, MO) for 6 h to detect the frequencies of IL-17 and IFN-γ-producing T cells in BD patients before and after CsA treatment. Brefeldin A (10 μg/ml; Sigma) was added to cultured PBMCs for 4 h. The stimulated PBMCs were washed in phosphate buffered saline (137 mM sodium chloride, 2.7 mM potassium chloride, 10 mM disodium hydrogen phosphate, 2 mM potassium dihydrogen phosphate, pH 7.4) and incubated with phycoerythrin (PE)-cy7-labeled anti-CD8 (eBioscience) and fluorescein isothiocyanate- labeled anti-CD69 (eBioscience) or matched isotype (eBioscience) for 30 min in the dark at 4 °C . These PBMCs were fixed in 4% formaldehyde, permeabilized with 0.1% saponin (Sigma), and stained with Percp-labeled anti-CD3 (BD PharMingen, San Diego, CA), PE-labeled anti-IL-17 (eBioscience), PE-labeled anti-IFN-γ (eBioscience), or matched isotype control mAb (eBioscience). Cells were analyzed using a FACS Calibur and CellQuest software (Becton Dickinson Biosciences, San Jose , CA).

For determination of the influence of CsA on the production of cytokines in vitro, PBMCs were stimulated with phorbol 12-myristate,13-acetate (PMA) and ionomycin for 6 h in the presence of 50 ng/ml CsA.

### Statistical analysis

Statistical analysis was performed using the Student *t* test. A level of p<0.05 was considered statistically significant.

## Results

### In vitro inhibition of cyclosporin A on the production of interleukin-17 and interferon-γ by peripheral blood mononuclear cells from patients with Behcet’s disease and normal controls

**Figure 1:**
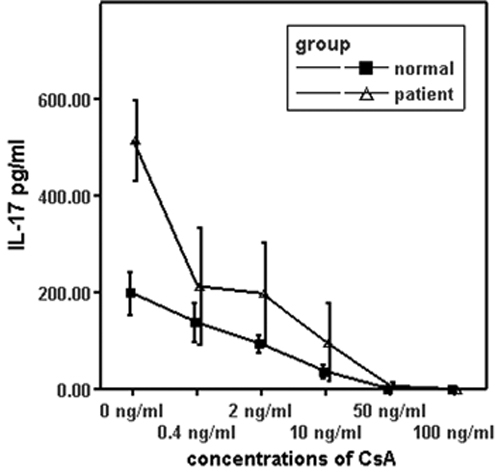
The effect of cyclosporin A on the production of interleukin-17 in vitro. Isolated peripheral blood mononuclear cells (1×10^6^ cells/ml) from BD patients with active uveitis and normal controls were stimulated with anti-CD3 and anti-CD28 antibodies in the presence of different concentrations of CsA for 72 h. The IL-17 level in the supernatant was detected using ELISA analysis. Data are the means±SD of triplicate samples.

**Figure 2:**
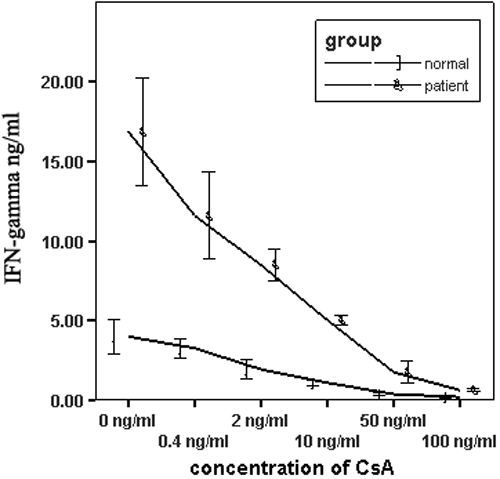
The effect of cyclosporin A on the production of interferon-γ in vitro. Isolated peripheral blood mononuclear cells (1×10^6^ cells/ml) from BD patients with active uveitis and normal controls were stimulated with anti-CD3 and anti-CD28 antibodies in the presence of different concentrations of CsA for 72 h. IFN-γ production in the supernatant was detected using ELISA analysis. Data are the means±SD of triplicate samples.

The production of IL-17 and IFN-γ was significantly increased upon stimulation with anti-CD3 and anti-CD28 antibodies both in BD patients (n=15) and normal controls (n=14). Anti-CD3- and anti-CD28-stimulated PBMCs produced a larger amount of IL-17 and IFN-γ in active BD patients compared to normal controls (both p<0.001; Figure 1 and Figure 2). PBMCs isolated from BD patients and normal controls were stimulated with anti-CD3 and CD28 antibodies in combination with different concentrations of CsA (0.4, 2, 10, 50, and 100 ng/ml). The results showed that CsA significantly inhibited IL-17 production by polyclonally stimulated PBMCs from both BD patients and normal controls in a dose-dependent manner (p<0.05). IL-17 was completely blocked by CsA at a concentration as low as 50 ng/ml (Figure 1 and Figure 2).

### In vitro influence of cyclosporin A on interleukin-17 and interferon-γ production by T cells and on T-cell activation

**Figure 3:**
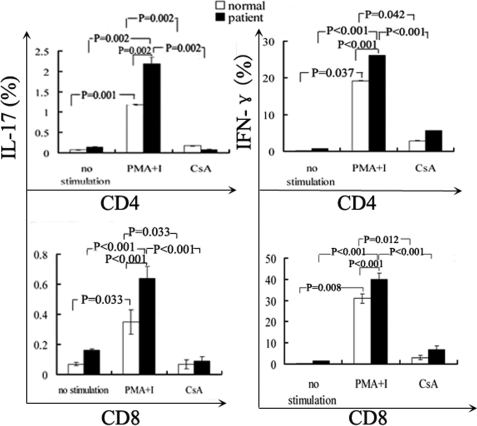
The effect of cyclosporin A on the percentages of interleukin-17-producing and interferon-γ-producing T cells from patients with Behcet’s disease and normal controls in vitro. Isolated peripheral blood mononuclear cells (1×10^6^ cells/ml) were stimulated with phorbol 12-myristate,13-acetate/ionomycin for 6 h in the absence or presence of CsA (50 ng/ml). The percentage of both IL-17-producing and IFN-γ-producing CD4^+^ or CD8^+^ T cells was significantly higher in BD patients than in normal controls and was significantly reduced in the presence of CsA. Data are expressed as means±SD.

**Figure 4:**
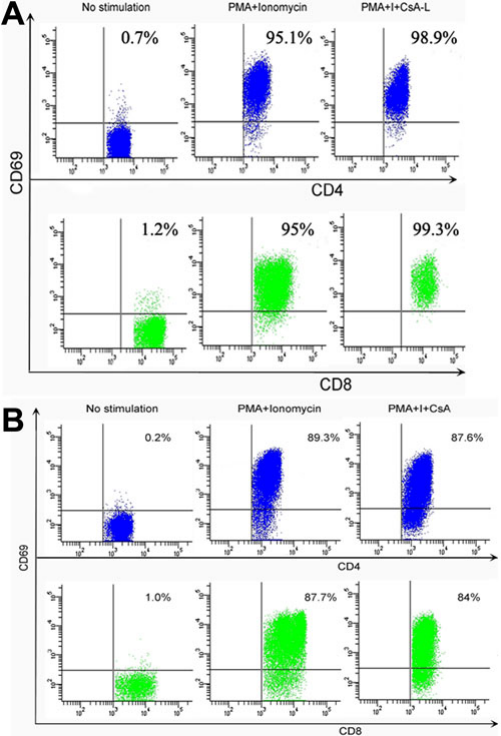
The effect of cyclosporin A on T- cell activation at a concentration of 50 ng/ml. Isolated peripheral blood mononuclear cells (1×10^6^ cells/ml) from normal controls and patients with Behcet’s disease were stimulated with PMA/ionomycin for 6 h in the presence of CsA (50 ng/ml). Cells were analyzed using FACS. The expression of CD69 by CD4^+^ and CD8^+^ T cells from (**A**) normal controls and (**B**) BD patients was not inhibited by CsA (50 ng/ml). Data are shown representative of five independent experiments.

Isolated PBMCs from BD patients and normal controls were stimulated with PMA (20 ng/ml) and ionomycin (1 µg/ml) for 6 h in the presence of CsA (50 ng/ml) to study the regulatory effect of CsA on IL-17 and IFN-γ production. The results showed that CsA could significantly lower the frequencies of IL-17 and IFN-γ in CD4^+^ and CD8^+^ T cells from both BD patients and normal controls (Figure 3). However, CsA did not influence the expression of CD69 in CD4^+^ and CD8^+^ T cells induced by PMA and ionomycin (Figure 4A,B).

### The influence of cyclosporin A on the production of interleukin-17 and interferon-γ by peripheral blood mononuclear cells from patients with Behcet’s disease in vivo

**Figure 5:**
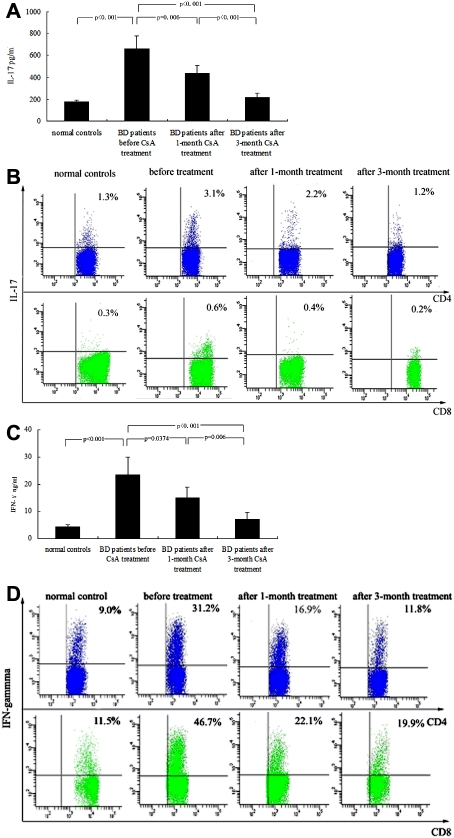
The production of interleukin-17 and interferon-γ by polyclonally stimulated peripheral blood mononuclear cells from patients with Behcet’s disease before and after one- and three- month cyclosporin A treatment. **A**: Levels of IL -17 in supernatants of peripheral blood mononuclear cells from BD patients (n=6) before and after treatment and normal controls (n=6) were determined by ELISA. IL-17 production by PBMCs was significantly downregulated in BD patients after CsA treatment compared with that before treatment. **B**: The frequency of IL-17-producing T cells from BD patients before and after treatment and normal controls was determined by FACS. Both IL-17-producing CD4^+^ and CD8^+^ T cells were significantly decreased in BD patients at 1 and 3 months after CsA treatment compared with those before treatment. **C**: Concentrations of IFN-γ in supernatants of PBMCs from BD patients before and after treatment and normal controls were determined by ELISA. IFN-γ production by polyclonally stimulated PBMCs was significantly lower in BD patients after CsA treatment than before treatment. **D**: The frequency of IFN-γ-producing T cells from BD patients before and after treatment and normal controls were determined by FACS. Both IFN-γ-producing CD4^+^ and CD8^+^ T cells were significantly decreased in BD patients after CsA treatment compared with those before treatment. Data are shown representative of five independent experiments.

The aforementioned study demonstrated that CsA could significantly suppress IL-17 and IFN-γ production by PBMCs in vitro. To detect the effect of CsA on IL-17 and IFN-γ production in vivo, six BD patients with active uveitis were treated by CsA and re-examined for these cytokines at 1 and 3 months after treatment. At 1 month after treatment with CsA, the intraocular inflammation in these six patients was markedly inhibited. A 3-month treatment with CsA almost completely and clinically resolved the intraocular inflammation in these patients. Both FACS and ELISA results showed that IL-17 production by stimulated PBMCs was significantly decreased after a 1-month treatment (p<0.001, p=0.006, respectively) and 3-month treatment (both p<0.001) as compared with production before treatment. The IL-17 production by PBMCs after a 3-month treatment was similar to that in normal controls (Figure 5A,B). The production of IFN-γ by stimulated PBMCs was also significantly suppressed in these patients after 1 and 3 months of CsA treatment in the ELISA experiment (p=0.037, p=0.006, respectively; Figure 5C). A similar result was observed in the FACS experiment (both p<0.001; Figure 5D).

## Discussion

In this study we investigated the effect of CsA on IL-17 and IFN-γ production in vitro and in vivo. An in vitro experiment showed that CsA suppressed the production of IL-17 and IFN-γ in BD patients with active uveitis. A 3-month treatment with this medicine significantly inhibited the production of IL-17 and IFN-γ in BD patients in association with amelioration of the intraocular inflammation. All these findings suggest that CsA may exert its regulative function possibly through suppressing IL-17 and IFN-γ production.

CsA has been shown to be effective in the treatment of BD and Vogt-Koyanagi-Harada syndrome [22-24]. In both diseases IL-17 has been considered as one of the important cytokines. Our recent studies showed that both CsA and corticosteroids exerted a therapeutic role through inhibiting production of IL-17 in VKH patients and that CsA had a much stronger inhibitory effect on IL-17 production compared to corticosteroids [18,24]. In this study, we found an increased IL-17 expression in association with active uveitis in BD, although the results did not show that it was directly involved in this disease. Another purpose of this study was to examine whether CsA could inhibit both BD activity and IL-17 production and whether there was a correlation between this cytokine and inflammatory activity. The results showed that CsA significantly suppressed intraocular inflammation at 1 month and almost resolved this inflammation clinically at 3 months following treatment. The IL-17 production by PBMCs was significantly decreased at 1 month and almost returned to a normal level at 3 months following treatment. The coincidence of downregulated expression of IL-17 and decreased BD activity seems to suggest a causative relationship between them. The previous unresponsiveness of these patients to corticosteroids suggests a predominant effect of CsA in our treatment for BD activity, although both medicines may have an additive effect on the production of IL-17. Additionally, an in vitro experiment showed that CsA alone could potently inhibit IL-17 production by PBMCs from BD patients in a dose-dependent manner. More importantly, it could completely block the IL-17 production at a relatively low dose. This result is consistent with that reported by Ziolkowska et al. [19]. They showed that CsA inhibited IL-17 produced by PBMCs stimulated with IL-15 and PMA.

IFN-γ is another important cytokine involved in BD and VKH syndrome [9,10,25,26]. Both CsA and corticosteroids have been demonstrated to downregulate this cytokine in VKH syndrome. In our study, we also investigated whether CsA inhibits the production of IFN-γ in BD patients in vivo and in vitro. The results also showed that CsA significantly inhibited in vitro production of IFN-γ, consistent with those reported previously in VKH syndrome [18,27]. The CsA treatment also inhibited the production of IFN-γ in BD patients in association with a striking amelioration of active intraocular inflammation. With prolonged treatment a stronger inhibition on both IFN-γ production and BD activity was observed. However, unlike the effect of CsA on IL-17, it did not entirely block the production of IFN-γ. This result is similar to that observed in the experiment with PBMCs from VKH syndrome patients [18]. In an earlier study on VKH syndrome, we found that CsA could also exert its role through inhibiting IL-17^+^ IFN-γ^+^ double-positive T cells. It is not clear whether there are increased IL-17^+^ IFN-γ^+^ T cells in BD and whether CsA could inhibit this population of T cells in this disease. More studies are needed to address these issues.

It is interesting to note a protective role of IL-17 in experimental autoimmune uveitis (EAU), a well-described counterpart in animals for human uveitis [28]. This result seems to be in conflict with that observed in human autoimmune diseases. The discrepancy between human diseases and animal models is not completely understood. Studies on this difference may greatly contribute to our understanding about BD physiologically and pathologically. It has been reported that γδ T cells are a potent source of IL-17 [29] and are involved in the development of EAU [30]. It is not yet clear whether this cell population plays a role in BD. More study is needed to clarify this issue.

CD69 is a T-cell-surface activation marker and has been shown to be increased in expression in BD [10,31]. It is not yet known whether CsA inhibits this molecular marker in BD. One of our aims was to address whether CsA could influence its expression in this disease. It is interesting to note that CsA did not influence CD69 expression at a concentration of 50 ng/ml in vitro using PMA/ionomycin as stimulators. This result is consistent with that reported earlier by Ortiz et al. [32] who found that CsA did not influence CD69 expression induced by PMA and TNF-α but decreased the expression of CD69 induced by IL-15 and a calcium ionophore. Further study is needed to clarify the influence of CsA on CD69 expression induced by these two stimulators in BD disease.

In conclusion, our study showed that CsA could completely block the in vitro production of IL-17 and significantly decrease the production of IFN-γ. A 3-month treatment of CsA greatly ameliorated the intraocular inflammation of BD patients in association with downregulated expression of IL-17 and IFN-γ. Together with our previous study on VKH disease, these results seem to suggest that CsA may exerts its therapeutic effect on BD possibly through inhibiting IL-17 and IFN-γ and that it may be universally used for the treatment of diseases mediated by IL-17 as well as IFN-γ. On the other hand, it is not clear if other medicines or agents used for the treatment of BD as well as other autoimmune diseases, for instance inflixmab and IFN-α, have effects similar to CsA. More studies are needed to clarify these issues.
